# Taraxasterol Inhibits LPS-Induced Inflammatory Response in BV2 Microglia Cells by Activating LXRα

**DOI:** 10.3389/fphar.2018.00278

**Published:** 2018-04-04

**Authors:** Bin Liu, Zhaoqi He, Jingjing Wang, Zhuoyuan Xin, Jiaxin Wang, Fan Li, Yunhe Fu

**Affiliations:** ^1^Cardiovascular Disease Center, First Hospital of Jilin University, Changchun, China; ^2^Department of Clinical Veterinary Medicine, College of Veterinary Medicine, Jilin University, Changchun, China; ^3^Department of Pathogenobiology, The Key Laboratory of Zoonosis, Chinese Ministry of Education, College of Basic Medicine, Jilin University, Changchun, China

**Keywords:** taraxasterol, LPS, ABCA1, LXRα, lipid rafts

## Abstract

Neuroinflammation plays a critical role in the development of neurodegenerative diseases. Taraxasterol, a pentacyclic-triterpene isolated from *Taraxacum officinale*, has been reported to have anti-inflammatory effect. The aim of this study was to investigate the anti-inflammatory effects and mechanism of taraxasterol in LPS-stimulated BV2 microglia cells. BV2 microglia cells were treated with taraxasterol 12 h before LPS stimulation. The effects of taraxasterol on LPS-induced TNF-α and IL-1β production were detected by ELISA. The effects of taraxasterol on LXRα, ABCA1, TLR4, and NF-κB expression were detected by western blot analysis. The results showed that taraxasterol dose-dependently inhibited LPS-induced TNF-α and IL-1β production and NF-κB activation. Taraxasterol also disrupted the formation of lipid rafts and inhibited translocation of TLR4 into lipid rafts. Furthermore, taraxasterol was found to activate LXRα-ABCA1 signaling pathway which induces cholesterol efflux from cells. In addition, our results showed that the anti-inflammatory effect of taraxasterol was attenuated by transfection with LXRα siRNA. In conclusion, these results suggested that taraxasterol inhibits LPS-induced inflammatory response in BV2 microglia cells by activating LXRα-ABCA1 signaling pathway.

## Introduction

Neuroinflammation, a chronic inflammation in the brain, has been reported to play critical roles in the development of neurodegenerative diseases, such as Alzheimer’s disease and Parkinson’s disease (Sun et al., 2010). Microglia, a type of primary immune cells in the brain, play critical roles in host defense and tissue repair in brain (Cameron and Landreth, 2010). Stimulation of microglia by LPS leads to the activation of TLR4 signaling pathway (Qin et al., 2005). The activation of TLR4 signaling pathway leads to the activation of NF-κB and release of inflammatory cytokines such as TNF-α and IL-1β (Kawai and Akira, 2007). TLR4 is the major receptor of LPS and inhibition of TLR4 signaling pathway could attenuate neurodegenerative diseases. Overproduction of these inflammatory cytokines leads to cell death and brain injury (Ziebell and Morganti-Kossmann, 2010). Therefore, the control of microglial activation could be a therapeutic approach for the treatment of neurodegenerative diseases.

Taraxasterol, a pentacyclic-triterpene isolated from *Taraxacum officinale*, has been reported to have anti-inflammatory effects (Xiong et al., 2014). Taraxasterol has been reported to inhibited iNOS and COX-2 expression in LPS-stimulated RAW264.7 cells (Xiong et al., 2014). Taraxasterol also inhibited IL-1β-induced NO and PGE2 production in human osteoarthritic chondrocytes (Piao et al., 2015). *In vivo*, taraxasterol was found to protect LPS-induced acute lung injury and endotoxic shock in mice (San et al., 2014; Zhang et al., 2014). Furthermore, taraxasterol has been reported to protect against OVA-induced allergic asthma in mice (Liu et al., 2013). However, the anti-inflammatory effects of taraxasterol on LPS-induced inflammatory response in BV2 microglia cells have not been reported. In addition, the anti-inflammatory mechanism of taraxasterol has not been fully clarified. In the present study, we detected the anti-inflammatory effects and mechanism of taraxasterol in LPS-stimulated BV2 microglia cells. Our results showed that taraxasterol inhibited LPS-induced inflammatory response in BV2 microglia cells by activating LXRα-ABCA1 signaling pathway.

## Materials and Methods

### Materials

Taraxasterol (purity: > 98%) was purchased from Chengdu Preferred Biotechnology Co., Ltd. (Chengdu, China). LPS (*Escherichia coli* O55:B5) and MTT was purchased from Sigma (St. Louis, MO, United States). Enzyme-linked immunosorbent assay (ELISA) kits of TNF-α and IL-1β were purchased from Biolegend (CA, United States). Antibodies against LXRα and ABCA1 monoclonal antibodies were purchased from Santa Cruz Biotechnology Inc. (Santa Cruz, CA, United States). Antibodies against TLR4, NF-κB p65, IκBα, and β-actin monoclonal antibodies were purchased from Cell Signaling Technology (Danvers, MA, United States). FuGENE HD transfection reagent was purchased from Roche Applied Science (Indianapolis, IN, United States).

### Cell Culture and Treatment

BV2 microglia cells were purchased from the Institute of Basic Medical Sciences of the China Science Academy. The cells were cultured in DMEM supplemented with 10% heat-inactivated fetal bovine serum at 37°C in a humidified incubator under 5% CO_2_. The cells were pretreated with taraxasterol (3, 6, 12 μg/ml) 12 h before LPS (0.5 μg/ml) treatment. The concentration of LPS used in this study was based on previous studies (Jeong et al., 2010; Wang et al., 2015).

### Effects of Taraxasterol on Cell Viability

The potential cytotoxicity of taraxasterol on BV2 microglia was evaluated by MTT assay as described previously (Yu et al., 2015). Briefly, BV2 microglia cells (2 × 10^5^ cells/ml) were seeded in 96 well plates and treated with different concentrations of taraxasterol and stimulated with LPS for 24 h. After the culture supernatants were removed, the resulting dark blue crystals were dissolved with DMSO. Absorbance was determined at 540 nm.

### Effects of Taraxasterol on LPS-Induced TNF-α and IL-1β Production

The effects of taraxasterol on LPS-induced inflammatory cytokines production were measured by ELISA as described previously (Yu et al., 2015). BV2 microglia cells were pretreated with taraxasterol for 12 h and stimulated with LPS for 24 h. The levels of inflammatory cytokines TNF-α and IL-1β were detected by ELISA (Biolegend, CA, United States) according to the manufacturer’s protocol.

### Effects of Taraxasterol on Cholesterol Levels in Lipid Rafts

Lipid rafts were isolated as described previously (Fu et al., 2014a). The level of cholesterol in lipid raft was assayed by gas–liquid chromatography as previously described (Fu et al., 2014a).

### Effects of Taraxasterol on Transcriptional Activity of LXRα

The effects of taraxasterol on transcriptional activity of LXRα were detected by LXR receptor gene assay as described previously (Fu et al., 2015). BV2 microglia were transfected with β-galactosidase control vector and LXRα luciferase reporter plasmid using FuGENE HD transfection reagent according to the manufacturer’s instructions. Six hours after transfection, cells were treated with taraxasterol for 12 h. Luciferase activity was normalized by β-galactosidase activity.

### Western Blot Analysis

Total proteins from cells were extracted by M-PER Mammalian Protein Extraction Reagent (Pierce, Rock ford, IL, United States). Protein concentrations were determined by BCA method. Equal amount of protein (30 μg) were loaded and electrophoresed on a 12% SDS–PAGE and transferred onto PVDF membrane (Millipore, Cork, Ireland). The membrane was blocked with 5% fetal bovine serum. Then the membrane was probed with primary antibodies overnight at 4°C. Subsequently, the membranes were probed with HRP-conjugated secondary antibodies for 2 h at room temperature. The immunoreactive bands were visualized using an enhanced chemiluminescence system (Thermo Scientific, United States). Density value of the bands was quantified using Quantity One Software (Bio-Rad, United States) and the values obtained were used for statistical analysis.

### LXRα siRNA Transfections

BV2 microglia cells were transfected with LXRα siRNA or control siRNA using FuGENE HD transfection reagent according to the manufacturer’s instructions (Fu et al., 2014b). 36 h later, the cells were treated with taraxasterol and stimulated with LPS. 24 h later, the levels of TNF-α and IL-1β were detected by ELISA.

### Statistical Analysis

All data were presented as means ± SEM of three independent experiments and analyzed using one-way ANOVA combined with Tukey’s multiple comparison tests. *P* < 0.05 was taken as statistically significant.

## Results

### Effects of Taraxasterol on Cell Viability

**FIGURE 1:**
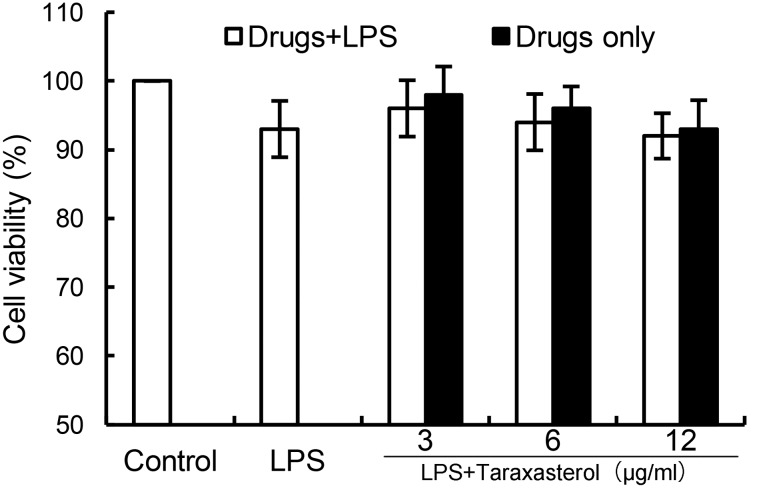
Effects of taraxasterol on the cell viability of BV2 microglia cells. BV2 microglia cells (2 × 10^5^ cells/ml) were seeded in 96 well plates and treated with different concentrations of taraxasterol and stimulated with LPS (0.5 μg/ml) for 24 h. The cell viability was determined by MTT assay. The values presented are the means ± SEM of three independent experiments.

As shown in **Figure 1**, the results showed that LPS (0.5 μg/ml) did not affect the cell viability of BV2 microglia. Taraxasterol at the concentration up to 12 μg/ml had no cellular toxicity on BV2 microglia (**Figure 1**).

### Effects of Taraxasterol on LPS-Induced TNF-α and IL-1β Production

**FIGURE 2:**
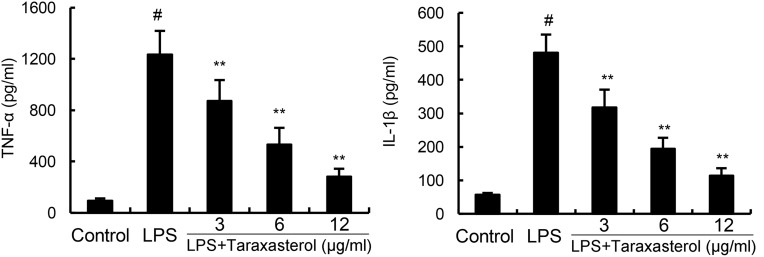
Effects of taraxasterol on LPS-induced TNF-α and IL-1β production. The data presented are the means ± SEM of three independent experiments. ^#^*p* < 0.05 vs. control group; ^∗^*p* < 0.05, ^∗∗^*p* < 0.01 vs. LPS group.

As shown in **Figure 2**, treatment of BV2 microglia with LPS resulted in significant increases in cytokines TNF-α and IL-1β production. However, taraxasterol significantly inhibited LPS-induced TNF-α and IL-1β production.

### Effects of Taraxasterol on LPS-Induced NF-κB Activation

**FIGURE 3:**
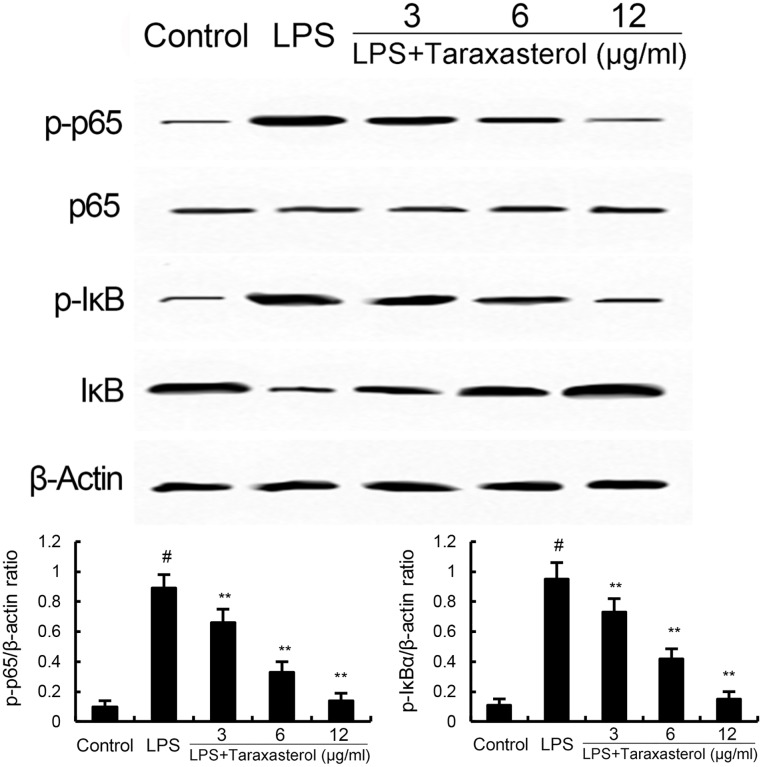
Taraxasterol inhibits LPS-induced NF-κB activation. The values presented are the means ± SEM of three independent experiments. ^#^*p* < 0.05 vs. control group; ^∗^*p* < 0.05, ^∗∗^*p* < 0.01 vs. LPS group.

The effects of taraxasterol on LPS-induced NF-κB activation were detected by Western blotting. As shown in **Figure 3**, LPS significantly up-regulated the expression of NF-κB. However, treatment of taraxasterol inhibited LPS-induced NF-κB activation in a dose-dependent manner.

### Effects of Taraxasterol on LPS-Induced TLR4 Translocation Into Lipid Rafts

**FIGURE 4:**
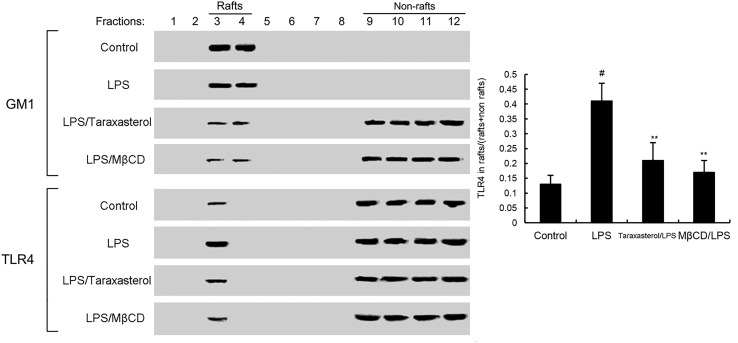
The recruitment of TLR4 to lipid rafts was inhibited by taraxasterol. Fractions 3–4 correspond to lipid rafts. Representative blots of three separate experiments are shown. TLR4 content of macrophage lipid rafts was calculated as a percentage of total membrane TLR4 (lipid rafts + non-rafts). The values presented are the means ± SEM of three independent experiments. ^#^*p* < 0.05 vs. control group; ^∗^*p* < 0.05, ^∗∗^*p* < 0.01 vs. LPS group.

As shown in **Figure 4**, treatment of cells with LPS induced the translocation of TLR4 into lipid rafts. However, taraxasterol significantly suppressed LPS-induced TLR4 translocation into lipid rafts.

### Taraxasterol Disrupts Lipid Rafts by Depleting Cholesterol

**FIGURE 5:**
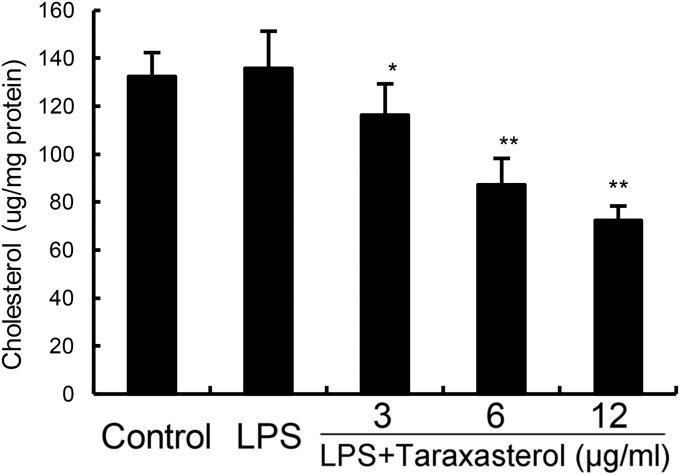
Effects of taraxasterol on lipid rafts cholesterol levels. Cells were treated with taraxasterol (3, 6, 12 μg/ml) for 12 h. Membrane cholesterol levels were measured by gas–liquid chromatography and the results were plotted as μg cholesterol/mg protein. The values presented are the means ± SEM of three independent experiments. ^#^*p* < 0.05 vs. control group; ^∗^*p* < 0.05, ^∗∗^*p* < 0.01 vs. LPS group.

As shown in **Figure 5**, taraxasterol significantly decreased the level of cholesterol in lipid rafts which results in the disrupting of lipid rafts.

### Cholesterol Replenishment Prevents the Anti-inflammatory Effects of Taraxasterol

**FIGURE 6:**
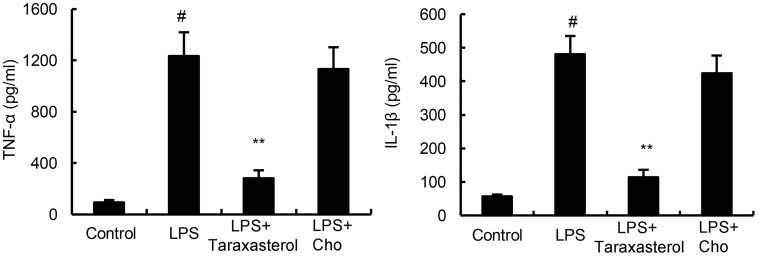
Cholesterol replenishment prevents the anti-inflammatory effect of taraxasterol. The values presented are the means ± SEM of three independent experiments. ^#^*p* < 0.05 vs. control group; ^∗^*p* < 0.05, ^∗∗^*p* < 0.01 vs. LPS group.

Cholesterol replenishment experiments were carried out in this study to investigate the effects of cholesterol in the anti-inflammatory mechanism of taraxasterol. The results showed that when cholesterol were added, the anti-inflammatory effects of taraxasterol were reversed (**Figure 6**).

### Effects of Taraxasterol on LXRα-ABCA1 Signaling Pathway

**FIGURE 7:**
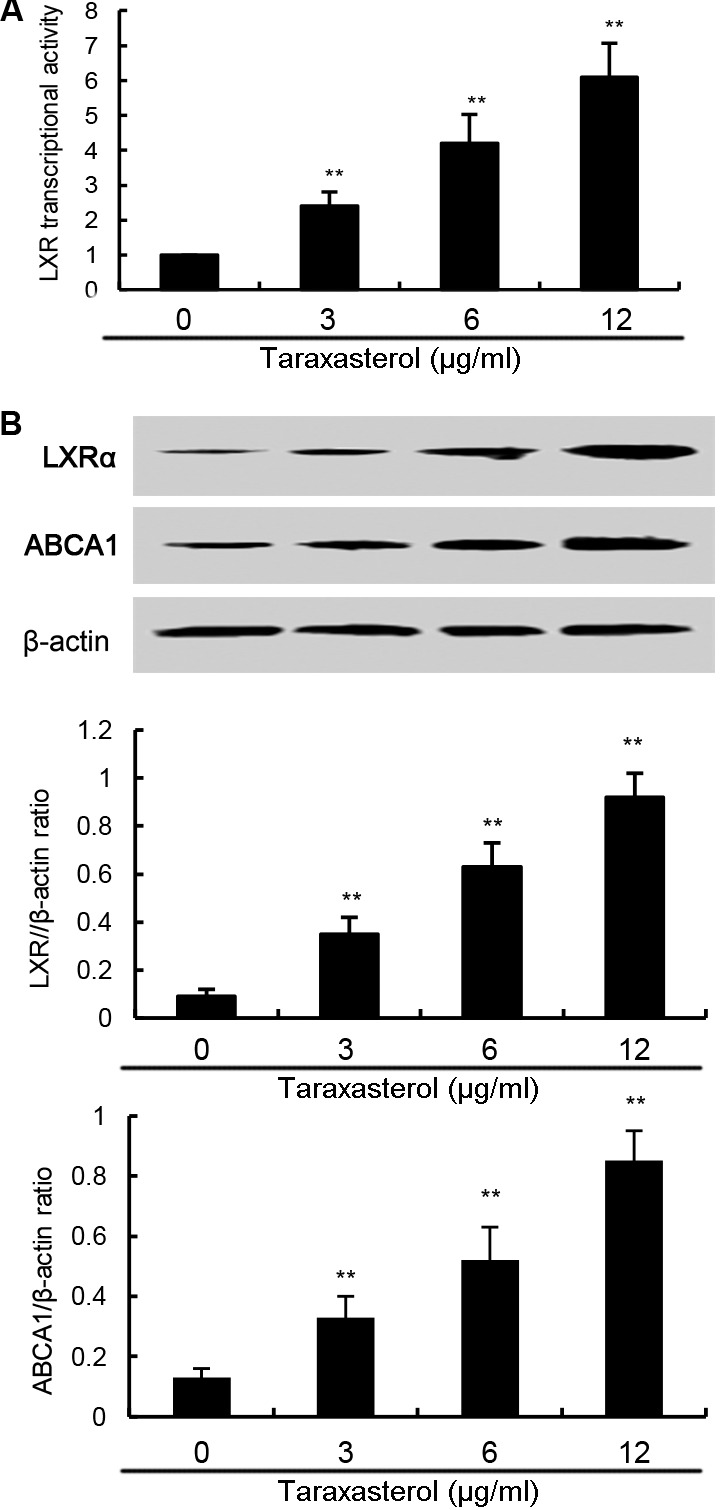
**(A)** Effects of taraxasterol on LXR transcriptional activity. **(B)** Effects of taraxasterol on LXRα and ABCA1 expression. The values presented are the means ± SEM of three independent experiments. ^#^*p* < 0.05 vs. control group; ^∗^*p* < 0.05, ^∗∗^*p* < 0.01 vs. LPS group.

In this study, the effects of taraxasterol on LXRα-ABCA1 signaling pathway were detected in this study. As shown in **Figure 7A**, taraxasterol significantly up-regulated the transcriptional activity of LXRα. Furthermore, taraxasterol was found to up-regulate the expression of LXRα and ABCA1 in a dose-dependent manner (**Figure 7B**).

### Taraxasterol Exerts Anti-inflammatory Activity Through Activating LXRα

**FIGURE 8:**
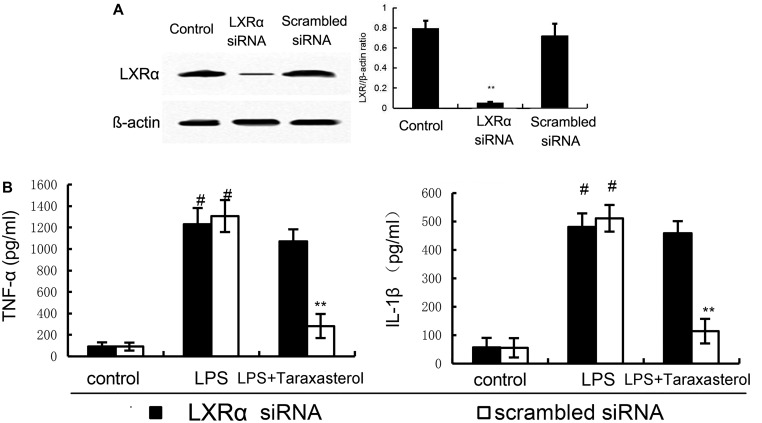
**(A)** The effects of siRNA on LXRα expression. **(B)** Knockdown of LXRα abrogated the effects of taraxasterol on LPS induces inflammatory response in BV2 microglia cells. The data presented are the means ± SEM of three independent experiments. ^#^*p* < 0.05 vs. control group; ^∗^*p* < 0.05, ^∗∗^*p* < 0.01 vs. LPS group.

To investigate whether activation of LXRα is responsible for the anti-inflammatory effect of taraxasterol, LXRα was knockdown by siRNA (**Figure 8A**). As shown in **Figure 8B**, the results showed that LXRα knockdown significantly reversed the inhibition of TNF-α and IL-1β production by taraxasterol.

## Discussion

Neurodegenerative diseases are accompanied by inflammation of the CNS (Amor et al., 2010). Studies showed that controlling of inflammation had the ability to treat neurodegenerative diseases (Gao et al., 2003; Carnevale et al., 2007). Taraxasterol, a pentacyclic-triterpene isolated from *T. officinale*, has been reported to have anti-inflammatory effects. In the present study, we investigated the effects of taraxasterol on LPS-induced inflammatory responses in BV2 microglia. The results showed that taraxasterol inhibited LPS-induced inflammatory cytokines production by activating LXRα-ABCA1 signaling pathway.

LPS, the main endotoxin produced by Gram-negative bacteria, has been identified as one of the most important factor that causes neurodegenerative diseases (Qin et al., 2007). Stimulating of microglia by LPS lead to the production of inflammatory cytokines such as TNF-α and IL-1ß (Brandenburg et al., 2010). These inflammatory cytokines initiate and amplify the inflammatory response and lead to development of neurodegenerative diseases (Lucas et al., 2006). Recent studies showed that inhibition of inflammatory cytokines production could attenuate the severity of neurodegenerative diseases (Arvin et al., 1996). In this study, our results showed that taraxasterol significantly inhibited LPS-induced inflammatory cytokines production. NF-κB is an important transcriptional factor that play a critical role in the regulation of TNF-α and IL-1ß production (Dong et al., 2010). Nowadays, NF-κB has been identified as the main target for the treatment of inflammatory diseases such as neurodegenerative diseases (Zipp and Aktas, 2006). In this study, our results showed that treatment of taraxasterol significantly inhibited LPS-induced NF-κB activation. These results suggested that taraxasterol inhibited LPS-induced inflammatory response by inhibiting NF-κB activation.

LPS stimulation induces TLR4 receptor dimerization and recruitment of TLR4 into lipid rafts, which subsequently induced the activation of NF-κB (Zhu et al., 2010). Lipid raft disruption leads to impairment in TLR4 signaling by preventing TLR4 translocation into lipid rafts (Fernandez-Lizarbe et al., 2008). To investigate the anti-inflammatory mechanism of taraxasterol, the effects of taraxasterol on LPS-induced recruitment of TLR4 into lipid rafts were detected by western blot analysis in this study. The results showed that taraxasterol significantly inhibited recruitment of TLR4 into lipid rafts. Furthermore, the effects of taraxasterol on cholesterol level in lipid rafts were detected in this study. Our results showed that taraxasterol disrupted the formation of lipid rafts by decreasing the level of cholesterol. These results suggested that taraxasterol disrupted the formation of lipid rafts by decreasing the level of cholesterol, thereby inhibited LPS-induced recruitment of TLR4 into lipid rafts and TLR4 signaling pathway.

The liver X receptors (LXRs) are members of the nuclear hormone receptor superfamily that are bound and activated by oxysterols (Lehmann et al., 1997). LXRα has previously been shown to regulate the metabolic conversion of cholesterol to bile acids (Peet et al., 1998). Activating of LXRα induces the expression of ABCA1, a lipid pump that effluxes cholesterol out of cells (Cavelier et al., 2006). Recent studies showed that many herbal compounds are the ligands of LXRα (Gong and Xie, 2004). In this study, our results showed that taraxasterol could activate LXRα and up-regulated the expression of LXRα and ABCA1, suggesting taraxasterol was a ligand of LXRα. To further confirm the anti-inflammatory mechanism of taraxasterol is through activating LXRα, LXRα was knockdown by siRNA. Our results showed that LXRα knockdown significantly reversed the inhibition of TNF-α and IL-1ß by taraxasterol. These results suggested that taraxasterol exerted anti-inflammatory effects via activating LXRα.

## Conclusion

These results showed that taraxasterol inhibited LPS-induced inflammatory response By activating LXRα-ABCA1 signaling pathway, which subsequently disrupting lipid rafts and inhibiting TLR4 translocation into lipid rafts, thereby inhibiting LPS-induced inflammatory responses. Taraxasterol might be a valuable agent for the treatment of neurodegenerative diseases.

## Author Contributions

YF and FL designed the experiments. BL, JW, ZX, and YF did the experiments. BL and YF wrote the paper. YF and ZH revised the paper.

## Conflict of Interest Statement

The authors declare that the research was conducted in the absence of any commercial or financial relationships that could be construed as a potential conflict of interest.
